# Burnout amongst neurosurgical trainees in the UK and Ireland

**DOI:** 10.1007/s00701-021-04873-5

**Published:** 2021-05-22

**Authors:** Nadia Liber Salloum, Phillip Correia Copley, Marco Mancuso-Marcello, John Emelifeonwu, Chandrasekaran Kaliaperumal

**Affiliations:** Department of Clinical Neurosciences, Royal Infirmary of Edinburgh, Little France Crescent, Edinburgh, EH16 4SA UK

**Keywords:** Burnout, Copenhagen Burnout Inventory, Neurosurgery training, Wellbeing

## Abstract

**Introduction:**

Burnout is becoming an increasingly recognised phenomenon within the medical profession. This study aims to investigate the presence of burnout amongst neurosurgical trainees in the UK and Ireland as well as investigating potential exacerbating and protective factors.

**Method:**

An online survey was sent to all neurosurgical trainees in the UK and Ireland via the British Neurosurgical Trainees’ Association (BNTA) mailing list. Responding participants anonymously completed the Copenhagen Burnout Inventory (CBI) and answered questions about known risk factors for burnout including workplace environment, workplace bullying, time spent on leisure activities and sleep and reported likelihood of leaving neurosurgery. We also collated data on responders’ demographics. We compared CBI scores for participants with and without risk factors to determine correlation with CBI.

**Results:**

There were 75 respondents (response rate 42%) from a range of ages and all training grades, 72% of whom were male. The median CBI score was 38.85 (IQR 17.76). Participants showed a higher degree of personal and workplace burnout (median CBIs of 47.02, IQR 25.00; and 49.14, IQR 19.64, respectively) compared with patient-related burnout (median CBI 18.67, IQR 25.00). Participants with the following self-reported risk factors were significantly more likely to have higher CBIs: workplace bullying (*p* = 0.01), getting on less well with colleagues (*p* < 0.05), working longer hours (*p* < 0.05) and insufficient sleep, exercise and leisure time (all *p* < 0.01). Those with higher CBI scores were more likely to consider leaving neurosurgical training (*p* = 0.01).

**Conclusion:**

We identified a high burnout incidence in a cohort representative of UK neurosurgical trainees, although our results may have been skewed somewhat by selection bias. We determined potential risk factors for burnout related to specific workplace stressors and time for non-work activities. In the future, changes to training curricula should address these issues, aiming to improve training, enhance patient care and reduce attrition rates.

**Supplementary Information:**

The online version contains supplementary material available at 10.1007/s00701-021-04873-5.

## Introduction

Burnout was initially introduced as a concept in the early 1970s by Freudenberger [6, 8]. Over the following decade, and with the input from others, including Maslach and Pines [6, 15], the concept was further refined. The syndrome described as ‘burnout’ was applied to professionals working in human services, often in care and healthcare environments, where there is a large volume of interaction with people [6, 8, 16]. As described by Maslach (1996), “burnout is a psychological syndrome of emotional exhaustion, depersonalisation, and reduced personal accomplishment that can occur among individuals who work with other people in some capacity…” [17].

In 1981, Maslach published the Maslach Burnout Inventory (MBI), a survey by which burnout could be assessed quantitatively [16, 17]. Over the years, this has been refined, and there are now several different versions of the test, each tailored to different groups [17]. Other scales have been developed to assess burnout in the last couple of decades such as the Copenhagen Burnout Inventory (CBI) [13] and the Professional Fulfillment Index [24]. The CBI divides assessments into subcategories based on potential causative or influential factors in the burnout experienced, providing an alternative perspective on the analysis of burnout assessed. In addition, the CBI has been found to be comparable in terms of its validity in accurately identifying burnout when compared to the MBI and other assessment tools [5, 27].

Today the recognised definition of burnout consists of the presence of three key factors: emotional exhaustion, depersonalisation and personal accomplishment [16, 18]. Burnout can lead to feelings of exhaustion, cynicism and result in individuals being less effective at work [18]. Whilst often considered separate from depression, it has been noted that there is overlap between the two and that individuals with evidence of burnout can eventually go on to develop depression if left unaddressed [3, 18, 20].

The presence of burnout has been widely studied amongst healthcare professionals, and a number of studies have found it to be common amongst doctors across a range of specialities [14]. Included in this have been a small handful of studies looking into the presence of burnout amongst neurosurgeons. A systematic review of these found the prevalence of burnout in neurosurgeons to be just under 50% [29]. Previous studies have shown that burnout is associated with negative individual psychological consequences and, on a systems-level, leads to increased medical errors and potential compromise of patient safety [21]. Moreover, the implication of burnout clinicians is that of increased cost to the health service, via inefficiency, errors leading to potential litigation, absenteeism and loss of healthcare professionals to other careers [21, 25].

This study aims to assess the prevalence of burnout amongst neurosurgical trainees working in the National Health Service (NHS) in the UK and Ireland and identify exacerbating and predictive factors for this syndrome.

## Methods

### Participants

Eligible participants in this study included all neurosurgical trainees currently working in neurosurgical departments across the UK and Ireland.

### Questionnaire

A modified Copenhagen Burnout Inventory (CBI) questionnaire was sent out to 180 neurosurgery trainees/residents across the UK and Ireland through the British Neurosurgical Trainees’ Association (BNTA). The modified questionnaire included questions on responders’ general demographics, medical education, home circumstances, work environment and time spent on sleep, physical exercise and leisure (Supplementary Material 1). These additional questions had answer options which were either categorical or ordinal, in the form of Likert scales. One question, on factors contributing to the possibility of leaving neurosurgery, had a free-text answer section. Informed consent was obtained prior to the commencement of the anonymous questionnaire. The questionnaire was open for a period of 6 weeks. Responses were collated anonymously.

### Statistical analysis

Following conclusion of the data collection period, results were analysed using R software, version 3.6.1 (2019–07-05). The data distribution was tested for normality using the Shapiro–Wilk test which determined that all further analyses carried out used non-parametric tests (Supplementary Material 2).

As per the CBI [13], burnout scores for each participant were calculated as the mean score in each of the three categories: personal, work-related and patient-related, as well as the mean overall score. The differences between these three subgroup scores were analysed using the Friedman test with Nemenyi post hoc analysis. Comparison of the overall mean CBI scores across different demographics was analysed using the Mann–Whitney *U* test or Kruskal–Wallis test as appropriate. The relationship between the mean overall CBI score and ordinal variables such as hours worked and likelihood of leaving neurosurgery was analysed using the Spearman correlation coefficient with confidence intervals calculated using Fisher *z*’ statistic as described by Fieller [7]. Statistical significance was defined as a *p*-value < 0.05. Qualitative free-text answers were analysed using categorical coding of themes using methods previously described [12].

## Results

A total of 75 neurosurgical trainees completed the questionnaire out of 180 trainees on the BNTA mailing list, providing a response rate of 42%. The demographics of the participants are summarised in Table 1.

**Table 1:** Demographics of study participants, overall numbers and percentages

		Number, *n* = 75	Percentage
Age	< *25*	1	1.3
	*25–29*	15	20.0
	*30–34*	29	38.7
	*35–39*	19	25.3
	*40–44*	6	8.0
	> *44*	5	6.7
Sex	*Female*	16	21.3
	*Male*	54	72.0
	*Not disclosed*	5	6.7
Ethnicity	*White*	41	54.7
	*Asian*	21	28.0
	*Black*	4	5.3
	*Mixed*	4	5.3
	*Other*	1	1.3
	*Not disclosed*	4	5.3
Marital status	*Single*	20	26.7
	*Married*	38	50.7
	*Other*	12	16.0
	*Separated*	1	1.3
	*Not disclosed*	4	5.3
Number of dependents	*0*	34	45.3
	*1*	21	28.0
	*2*	13	17.3
	*3*	4	5.3
	*Not disclosed*	3	4.0
Reside in same region as family	*Yes*	49	65.3
	*No*	16	21.3
	*N/A*	7	9.3
	*Not disclosed*	3	4.0
Country of birth	*UK*	47	62.7
	*Ireland*	2	2.7
	*EU*	5	6.7
	*Other*	15	20.0
	*Not disclosed*	6	8.0
Country of medical degree	*UK*	57	76.0
	*Ireland*	4	5.3
	*EU*	3	4.0
	*Other*	5	6.7
	*Not disclosed*	6	8.0
Stage of training	*ST1*	7	9.3
	*ST2*	9	12.0
	*ST3*	12	16.0
	*ST4*	8	10.7
	*ST5*	11	14.7
	*ST6*	3	4.0
	*ST7*	8	10.7
	*ST8*	5	6.7
	*Other*	11	14.7
	*Not disclosed*	1	1.3

**Fig. 1:**
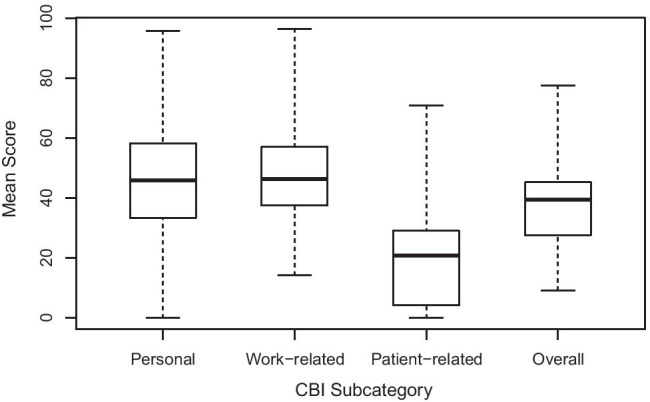
Box plot showing median, range and interquartile range for each of the CBI average subcategory scores across all respondents

The CBI results are summarised in Fig. 1 as the average scores for each subcategory: personal, work-related and patient-related, as well as the overall average score. A Friedman rank sum test showed that the patient-related score was significantly lower than both the personal (*p* < 0.001) and work-related (*p* < 0.001) scores, but there was no difference between the personal and work-related scores (*p* = 0.37).

Burnout scores were found to be significantly higher in those who have not taken time out of training (*p* = 0.023), those bullied in the workplace (*p* = 0.01) and those who have children or other dependents (*p* = 0.049). Those with high burnout scores were also more likely to have considered leaving neurosurgery either to retrain in another speciality (*p* = 0.01) or to leave medicine altogether (*p* = 0.011) (Supplementary Material 4). A positive correlation was identified between higher overall CBI scores and the following:

1. Higher average hours worked (*r*_s_ (75) = 0.27, *p* = 0.018)
2. Being negatively affected by patient outcomes (*r*_s_ (75) = 0.44, *p* < 0.001)
3. Likelihood of leaving neurosurgery (*r*_s_ (75) = 0.35, *p* = 0.002)

**Fig. 2:**
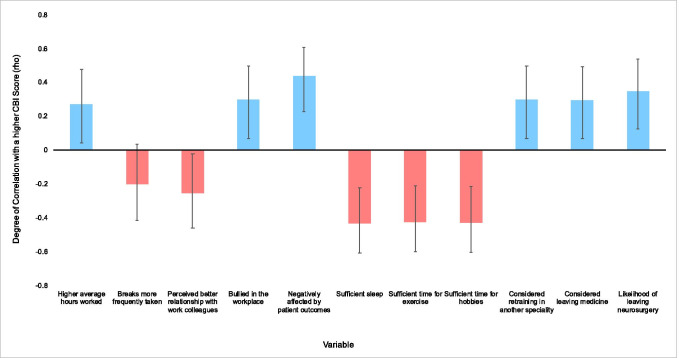
Correlation between tested variables and overall CBI score with 95% confidence intervals

There was a negative correlation between CBI score and positive colleague relationships (*r*_s_ (75) =  − 0.25, *p* = 0.029), self-reported insufficient time for sleep (*r*_s_ (75) =  − 0.43, *p* < 0.001), exercise (*r*_s_ (75) =  − 0.42, *p* < 0.001) and hobbies (*r*_s_ (75) =  − 0.43, *p* < 0.001) outwith work (Fig. 2, Supplementary Materials 3, 4, 5). There were no significant differences or correlation noted between CBI scores and any of the remaining variables tested.

Frequently reported reasons participants reported in the free-text answer section (total responses *n* = 45) for considering leaving neurosurgery were a poor work–life balance and long work hours (*n* = 12, 44%), a workplace culture of disrespect, bullying, harassment and undermining (*n* = 8, 18%), a lack of appreciation of their role as a trainee (*n* = 7, 16%), limited training opportunities due to short-staffing issues (*n* = 7, 16%), poor job prospects post-training (*n* = 6, 13%), limited work flexibility (*n* = 3, 7%) and lack of time available for academic endeavours (*n* = 3, 7%).

## Discussion

This is the first study looking into burnout amongst neurosurgical trainees in the UK and Ireland. Key correlations are identified between higher burnout scores and perception of workplace bullying, poor relationships with colleagues, higher average time spent at work and lack of time for sleep, exercise and hobbies. Findings also suggest that higher levels of burnout perhaps leave individuals more vulnerable to the psychological impact of challenging situations or outcomes at work. Those with higher scores were more likely to have considered other careers and be actively considering leaving neurosurgery, with the above factors frequently reported as reasons for considering this. Interestingly, the patient-related subcategory of burnout assessment most had a much lower score compared to the other two categories, further suggesting it is not the patients themselves which are contributing to burnout, but rather other aspects of the job.

Additionally, there were no correlations found between burnout levels and age, sex, ethnicity, marital status, country of medical education or stage of training, suggesting that the factors predisposing to burnout are inherent to neurosurgery training. This is perhaps somewhat surprising, particularly in relation to the lack of gender differences. Several studies have suggested that women are more likely to experience microaggressions in the workplace [23], undermining related to gender and the impact of family on their career [1]. A recent paper investigating burnout amongst surgical trainees in the USA revealed gender and racial abuse and mistreatment which was associated with increased burnout in women as a result of the mistreatment [10]. This was not borne out by our study.

In contrast to previous studies based in the USA and China [29], the participants in this study all work in a public health system, the National Health Service (NHS), rather than private- or insurance-based systems. Furthermore, other studies used the MBI questionnaires; therefore, whilst it is not possible to directly compare the burnout scores in our study to those previous studies, it is interesting that similar factors have been found to be associated with higher levels of burnout. Work-related issues such as bullying or a hostile work environment and high workloads with a poor work–life balance are common themes shared by this and previous studies and appear to play a greater part than other personal factors [2, 29]. Other areas raised in this study, such as perceived limited training opportunities and feelings of being underappreciated, are also similar to previously reported papers [29]. Although there are currently limited studies to compare average CBI scores, it is noted that the median scores in this study are higher than those found overall amongst hospital doctors including amongst physicians and general surgeons [13]. Overall, the results here appear to reflect findings of previous studies that the syndrome of ‘burnout’ pervades the medical profession and neurosurgeons are not immune to this phenomenon, as seems to be the case across a multitude of surgical specialities [2, 21, 22, 29]. Moreover, our study highlights both contributory and protective factors that should be addressed to mitigate the effects of burnout on trainees.

On a system level, our results clearly show that healthcare systems should target bullying and hostile working environments. Furthermore, it can be argued that trainees should not be forced to work outside of their rostered hours in order to minimise the effects of burnout. Whilst there is a general appreciation that the traditional surgical training environment which stereotypically allowed bullying is not a healthy working environment [11, 19, 26], this study illustrates that considerably more needs to be done on a system and departmental level to solve this problem. It is anticipated that by improving this, the more positive environment and reduced burnout rates will result in fewer staff absences, a lower attrition rate of trainees leaving the programme, alongside improvements in both economic and patient safety-related measures [21].

Additional strategies to address high levels of burnout could include providing support systems or wellbeing programmes which provide strategies to help cope with the challenges of a demanding workload and to provide robust systems by which issues can be raised if there are concerns amongst trainees either within the department or on a higher level if necessary. Indeed, previous studies where a ‘wellness programme’ has been trialled have shown provisional results, suggesting this may help with overall health and wellbeing amongst neurosurgeons [28]. This study correctly noted, however, that there is no ‘one option that fits all’ and that different individuals will have different needs. Identifying the individual trainees’ needs in order to support them through training is key. Other changes which improve the autonomy and flexibility of work and training include improved ease of access to less than full-time training and involving trainees in the construction of working patterns. This may allow individuals to tailor work to their individual needs and, therefore, help improve wellbeing and reduce levels of burnout. In the UK, there is no formal provision for psychological counselling or support for doctors who often face challenging situations both in and out of work, and evidence shows that such support may be beneficial in preventing burnout and subsequently depression, absenteeism and a change in career choice [4, 21]. The ability to identify burnout at an early stage and robust support mechanisms within a surgical department may help curb such consequences.

This study captured the responses of neurosurgical trainees at various stages of training in the UK and Ireland who work in a public health system. It is noted that this study assessed 75 trainees, representing 42% of those on the BNTA mailing and 30% of the 254 neurosurgical trainees currently listed on the UK’s General Medical Council (GMC) register [9]. Demographically, participants in this study were not dissimilar to the population of current GMC registered neurosurgical trainees, of which 61 (24%) are female and 116 (46%) fall within the 30–34 age category, the most common age category [9]. Therefore, although the findings cannot be generalised to every neurosurgical trainee, they are likely representative and capture many of the general themes and attitudes expressed by those in the speciality.

A limitation of the study is the ‘snapshot’ impression it gives, reflecting only the opinions at the time of the questionnaire rather than over a longer period of time. It has been previously noted that CBI scores are dynamic and can change depending on current circumstances [13]. Of particular note, with regard to the timing of this questionnaire, the COVID-19 pandemic has had a massive impact on work practices. Specifically, there has been a reduction in both elective and emergency neurosurgical operating across the country and a change in working patterns affecting trainees differently according to region. This may have not only an impact on the work environment, but also the overall wellbeing of trainees compared to previous years. There may have been a selection bias created by those who responded to the questionnaire who may have had a tendency to be experiencing either more or less burnout that the overall population of neurosurgical trainees. Responses were also limited to the scope of the questions asked, the majority offering categorical answer options, limiting the ability for individuals to express content outwith this. This did, however, allow the quantification of responses in order to better assess the situation and provide data to build a profile of the prevalence of burnout in the cohort. Moreover, free-text response questions were used to mitigate this limitation, with qualitative assessment of these answers in a standardised fashion.

Future studies should focus on assessing burnout across different healthcare settings and countries in order to identify whether certain environments/systems are more conducive to a healthy workplace. Studying the levels of burnout over time and following the introduction of measures outlined above will be invaluable in guiding ongoing progress in tackling burnout in neurosurgery and more generally in the medical profession.

## Conclusion

Burnout is clearly a major issue amongst neurosurgical trainees in the UK the exact prevalence identified here may be somewhat skewed by selection bias. Here, several correlating factors are identified and provide targets for future efforts to reduce the prevalence of burnout amongst neurosurgeons. In particular, improving the provision of a positive work environment and providing options to improve work–life balance are likely to have the largest impact in reducing burnout. In addition to improving individual wellbeing, this will likely also encourage trainees to remain in the profession and reduce the cost burden to the health system, through reducing the expense of sick leave and unused training. Ultimately burnout can lead to serious medical errors, and striving for improvement in patient safety is paramount, especially in a profession where the costs of such errors may have an immense effect on the patient and their families.

### Supplementary Information

Below is the link to the electronic supplementary material.

Supplementary file1 (DOCX 214 KB)
