# Supplementary material for: Burnout amongst neurosurgical trainees in the UK and Ireland
Source: Acta Neurochir (Wien). 2021 May 22;163(9):2383–9. doi: 10.1007/s00701-021-04873-5 (PMC8140310; doi:10.1007/s00701-021-04873-5)

**Supplementary Material**

**Article details:**

**Title:**

Burnout amongst neurosurgical trainees in the United Kingdom

**Authors:**

Nadia Liber Salloum

Phillip Correia Copley

Marco Mancuso-Marcello

John Emelifeonwu

Chandrasekaran Kaliaperumal

**Affiliation**

Department of Clinical Neurosciences, Royal Infirmary of Edinburgh, Edinburgh, UK

**Corresponding author**

Nadia Liber Salloum

Department of Clinical Neurosciences, Royal Infirmary of Edinburgh, Little France Crescent, Edinburgh, EH16 4SA, UK

nadialibersalloum@gmail.com

**Supplementary Material 1 - Survey Questions:**

Please select your age

- <25
- 25-29
- 30-34
- 35-39
- 40-44
- >44

Please select your gender

- Female
- Male
- Other
- Prefer not to say

Please select your ethnicity

- White
- Asian
- Black/African/Caribbean
- Mixed
- Other
- Prefer not to say

Please select the option that best reflects your marital status:

- Single
- Married or civil partnership
- Separated
- Divorced
- Widowed
- Other
- Prefer not to say

How many dependents do you have?

- 0
- 1
- 2
- 3
- 4
- 5
- >5
- Prefer not to say

Does your partner and/or dependent(s) live in the same city/region you currently work in?

- Yes
- No
- I do not have a partner/dependent(s)
- Prefer not to say

Please select where you were born

- UK
- Ireland
- EU/EEA
- Prefer not to say
- Other

Where did you complete your medical degree?

- UK
- Ireland
- EU/EEA
- Prefer not to say
- Other

Please select your current stage of neurosurgical training

- ST1
- ST2
- ST3
- ST4
- ST5
- ST6
- ST7
- ST8
- Other

Have you taken any time out of training?

If so, for what? Please select all options that apply

- No time taken out of training
- Additional degree (i.e. Masters, MD, PhD)
- Fellowship
- Locum work
- Travel
- Other

Are you on an academic training pathway?

- Yes
- No

How many hours do you work a week on average?

- <40
- 40-48
- 49-56
- >56

How often you get all your scheduled breaks without interruption?

- Never/almost never
- Seldom
- Sometimes
- Often
- Always

Do you feel worn out at the end of the working day?

- Never/almost never
- Seldom
- Sometimes
- Often
- Always

Do you feel that every working hour is tiring for you?

- Never/almost never
- Seldom
- Sometimes
- Often
- Always

How often do you feel tired?

- Never/almost never
- Seldom
- Sometimes
- Often
- Always

How often do you feel worn out?

- Never/almost never
- Seldom
- Sometimes
- Often
- Always

Are you exhausted in the morning at the thought of another day at work?

- Never/almost never
- Seldom
- Sometimes
- Often
- Always

How often are you physically exhausted?

- Never/almost never
- Seldom
- Sometimes
- Often
- Always

How often are you emotionally exhausted?

- Never/almost never
- Seldom
- Sometimes
- Often
- Always

Is your work emotionally exhausting?

- To a very high degree
- To a high degree
- Somewhat
- To a low degree
- To a very low degree

How often do you feel weak and susceptible to illness?

- Never/almost never
- Seldom
- Sometimes
- Often
- Always

How often do you think “I can’t take this anymore”?

- Never/almost never
- Seldom
- Sometimes
- Often
- Always

Do you feel burnt out because of your work?

- To a very high degree
- To a high degree
- Somewhat
- To a low degree
- To a very low degree

Does your work frustrate you?

- To a very high degree
- To a high degree
- Somewhat
- To a low degree
- To a very low degree

Do you feel you get along with your fellow work colleagues?

- Never/almost never
- Seldom
- Sometimes
- Often
- Always

Have you ever felt bullied in the workplace?

- Yes
- No

Do you find it hard to work with patients?

- To a very high degree
- To a high degree
- Somewhat
- To a low degree
- To a very low degree

Do you find it frustrating to work with patients?

- To a very high degree
- To a high degree
- Somewhat
- To a low degree
- To a very low degree

Does it drain your energy to work with patients?

- To a very high degree
- To a high degree
- Somewhat
- To a low degree
- To a very low degree

Do you feel that you give more than get back when you work with patients?

- To a very high degree
- To a high degree
- Somewhat
- To a low degree
- To a very low degree

Are you tired of working with patients?

- Never/almost never
- Seldom
- Sometimes
- Often
- Always

Do you sometimes wonder how long you will be able to continue working with patients?

- Never/almost never
- Seldom
- Sometimes
- Often
- Always

How often do you feel negatively affected by patient outcomes?

- Never/almost never
- Seldom
- Sometimes
- Often
- Always

Have you ever considered retraining in a different speciality?

- Yes
- No

Have you ever considered leaving medicine to pursue a different career?

- Yes
- No

If you have considered a different career (different speciality or non-medical career), how likely are you to leave neurosurgery?

- Very likely
- Likely
- Somewhat likely
- Unlikely
- Very unlikely
- I have not considered leaving neurosurgery

If you have considered a different career (different speciality or non-medical career), what factors have led to this?

- Free text answer

How often do you feel you get adequate sleep?

- Never/almost never
- Seldom
- Sometimes
- Often
- Always

To what extent do you feel you are able to spend adequate time on exercise per week?

- Never/almost never
- Seldom
- Sometimes
- Often
- Always

To what extent do you feel you are able to spend adequate time on other hobbies/leisure activities per week?

- Never/almost never
- Seldom
- Sometimes
- Often
- Always

Do you have enough energy for family and friends during leisure time?

- Never/almost never
- Seldom
- Sometimes
- Often
- Always

**Supplementary Material 2: Assessment of normal distribution of the data**

- Shapiro-Wilk normality test
  - W = 0.96454, p-value = 0.03388


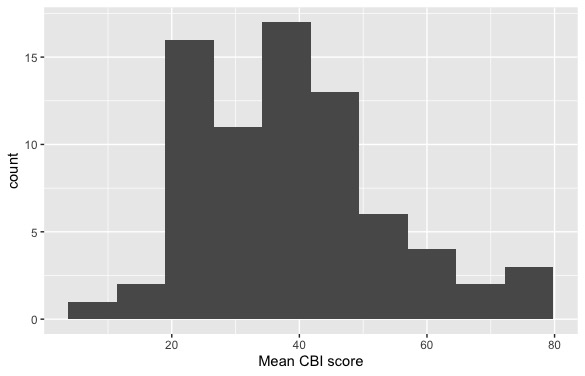


**Supplementary Material 3: Raw data for tested variables (excluding demographics)**

|  |  | **Total Number (Percentage)** |
| --- | --- | --- |
| Time out of Training | *No* | 44 (58.7) |
|  | *Yes* | 31 (41.3) |
| Academic Pathway | *No* | 65 (86.7) |
|  | *Yes* | 10 (13.3) |
| Average Hours Worked | *<40* | 1 (1.3) |
|  | *40-48* | 18 (24.0) |
|  | *49-56* | 23 (30.7) |
|  | *>56* | 33 (44.0) |
| Breaks Taken | *Never* | 27 (36.0) |
|  | *Seldom* | 26 (34.7) |
|  | *Sometimes* | 16 (21.3) |
|  | *Often* | 5 (6.7) |
|  | *Always* | 1 (1.3) |
| Get Along with Work Colleagues | *Seldom* | 3 (4.0) |
|  | *Sometimes* | 14 (18.7) |
|  | *Often* | 44 (58.7) |
|  | *Always* | 14 (18.7) |
| Bullied in the Workplace | *No* | 36 (48.0) |
|  | *Yes* | 39 (52.0) |
| Negatively Affected by Patient Outcomes | *Never* | 5 (6.7) |
|  | *Seldom* | 19 (25.3) |
|  | *Sometimes* | 42 (56.0) |
|  | *Often* | 6 (8.0) |
|  | *Always* | 3 (4.0) |
| Considered Retraining in Another Speciality | *No* | 40 (53.3) |
|  | *Yes* | 35 (46.7) |
| Considered Leaving Medicine | *No* | 32 (42.7) |
|  | *Yes* | 43 (57.3) |
| Likelihood of Leaving Neurosurgery | *N/A* | 24 (32.0) |
|  | *Very Unlikely* | 20 (26.7) |
|  | *Unlikely* | 11 (14.7) |
|  | *Somewhat likely* | 15 (20.0) |
|  | *Likely* | 2 (2.7) |
|  | *Very Likely* | 3 (4.0) |
| Sufficient Sleep | *Never* | 6 (8.0) |
|  | *Seldom* | 26 (34.7) |
|  | *Sometimes* | 22 (29.3) |
|  | *Often* | 21 (28.0) |
| Sufficient Time for Exercise | *Never* | 21 (28.0) |
|  | *Seldom* | 22 (29.3) |
|  | *Sometimes* | 22 (29.3) |
|  | *Often* | 6 (8.0) |
|  | *Always* | 4 (5.3) |
| Sufficient Time for Hobbies | *Never* | 17 (22.7) |
|  | *Seldom* | 32 (42.7) |
|  | *Sometimes* | 21 (28.0) |
|  | *Often* | 5 (6.7) |

**Supplementary Material 4: Burnout score variation in across different participant variables**

| **Variable** | **Range** | **Median overall CBI score (IQR)** | **Statistical test** | **p-value** |
| --- | --- | --- | --- | --- |
| ***Demographics*** |  |  |  |  |
| Age | *25-29*  *30-34*  *35-39*  *40-44*  *>44* | 28.9 (15.1)  40.8 (17.1)  38.2 (28.3)  37.5 (7.3)  43.4 (32.9) | Kruskal-Wallis | 0.322 |
| Sex | *Male*  *Female* | 39.5 (22.7)  32.9 (12.2) | Mann-Whitney U test | 0.260 |
| Ethnicity | *White*  *Asian*  *Black*  *Mixed* | 39.5 (17.1)  38.2 (13.2)  40.8 (6.9)  28.3 (29.9) | Kruskal-Wallis | 0.849 |
| Marital status | *Single*  *Married*  *Other* | 28.9 (17.8)  40.8 (13.8)  37.5 (13.5) | Kruskal-Wallis | 0.119 |
| Dependents | *0*  *1*  *2*  *3* | 34.9 (18.4)  40.8 (17.1)  38.2 (21.1)  49.3 (18.8) | Kruskal-Wallis | 0.049 |
| Reside in the same region as family | *Yes*  *No* | 39.5 (14.5)  40.4 (22.4) | Mann-Whitney U test | 0.493 |
| Country of birth | *UK*  *Ireland*  *EU*  *Other* | 39.5 (17.8)  31.6 (11.8)  25.0 (6.6)  38.2 (12.5) | Kruskal-Wallis | 0.170 |
| Country of medical degree | *UK*  *Ireland*  *EU*  *Other* | 39.5 (15.8)  35.5 (24.3)  25.0 (3.3)  40.8 (6.1) | Kruskal-Wallis | 0.193 |
| ***Training*** |  |  |  |  |
| Stage of training | *ST1*  *ST2*  *ST3*  *ST4*  *ST5*  *ST6*  *ST7*  *ST8*  *Other* | 28.9 (19.7)  31.6 (15.8)  40.1 (13.8)  42.1 (18.4)  28.9 (20.4)  42.1 (5.3)  31.6 (15.5)  52.6 (18.4)  40.0 (30.9) | Kruskal-Wallis | 0.193 |
| Time out of training | *Yes*  *No* | 31.6 (17.1)  40.8 (17.1) | Mann-Whitney U test | 0.023 |
| Academic pathway | *Yes*  *No* | 40.8 (8.9)  39.5 (19.7) | Mann-Whitney U test | 0.527 |
| ***Working environment*** |  |  |  |  |
| Average hours worked | *Range from <40 to >56* | 39.5 (17.8) | Spearman correlation r_s_(75)=0.27 | 0.018 |
| Breaks taken | *Likert scale (5pt; Never – Always)* | 39.5 (17.8) | Spearman correlation r_s_(75)=-0.20 | 0.084 |
| Get along with work colleagues | *Likert scale (5pt; Never – Always)* | 39.5 (17.8) | Spearman correlation r_s_(75)=-0.25 | 0.029 |
| Bullied in the workplace | *Yes*  *No* | 40.8 (21.1)  34.2 (17.1) | Mann Whitney U test | 0.010 |
| Negatively affected by patient outcomes | *Likert scale (5pt; Never – Always)* | 39.5 (17.8) | Spearman correlation r_s_(75)=0.44 | <0.001 |
| Considered retraining in another speciality | *Yes*  *No* | 40.8 (17.1)  34.9 (18.4) | Mann Whitney U test | 0.010 |
| Considered leaving medicine | *Yes*  *No* | 40.8 (13.8)  30.9 (16.4) | Mann Whitney U test | 0.011 |
| Likelihood of leaving neurosurgery | *Likert scale (5pt; Very Unlikely – Very Likely)* | 39.5 (17.8) | Spearman correlation r_s_(75)=0.35 | 0.002 |
| ***Out-with work*** |  |  |  |  |
| Sufficient sleep | *Likert scale (5pt; Never – Always)* | 39.5 (17.8) | Spearman correlation r_s_(75)=-0.43 | <0.001 |
| Sufficient time for exercise | *Likert scale (5pt; Never – Always)* | 39.5 (17.8) | Spearman correlation r_s_(75)=-0.42 | <0.001 |
| Sufficient time for hobbies | *Likert scale (5pt; Never – Always)* | 39.5 (17.8) | Spearman correlation r_s_(75)=-0.43 | <0.001 |

**Supplementary Material 5: Correlation between variables and mean CBI scores (a) average hours worked, (b) self-perceived relationship with work colleagues, (c) perception of being negatively affected by patient outcomes, (d) likelihood of leaving neurosurgery, (e-g) self reported access to adequate: sleep (e), exercise (f), and hobbies (g).**


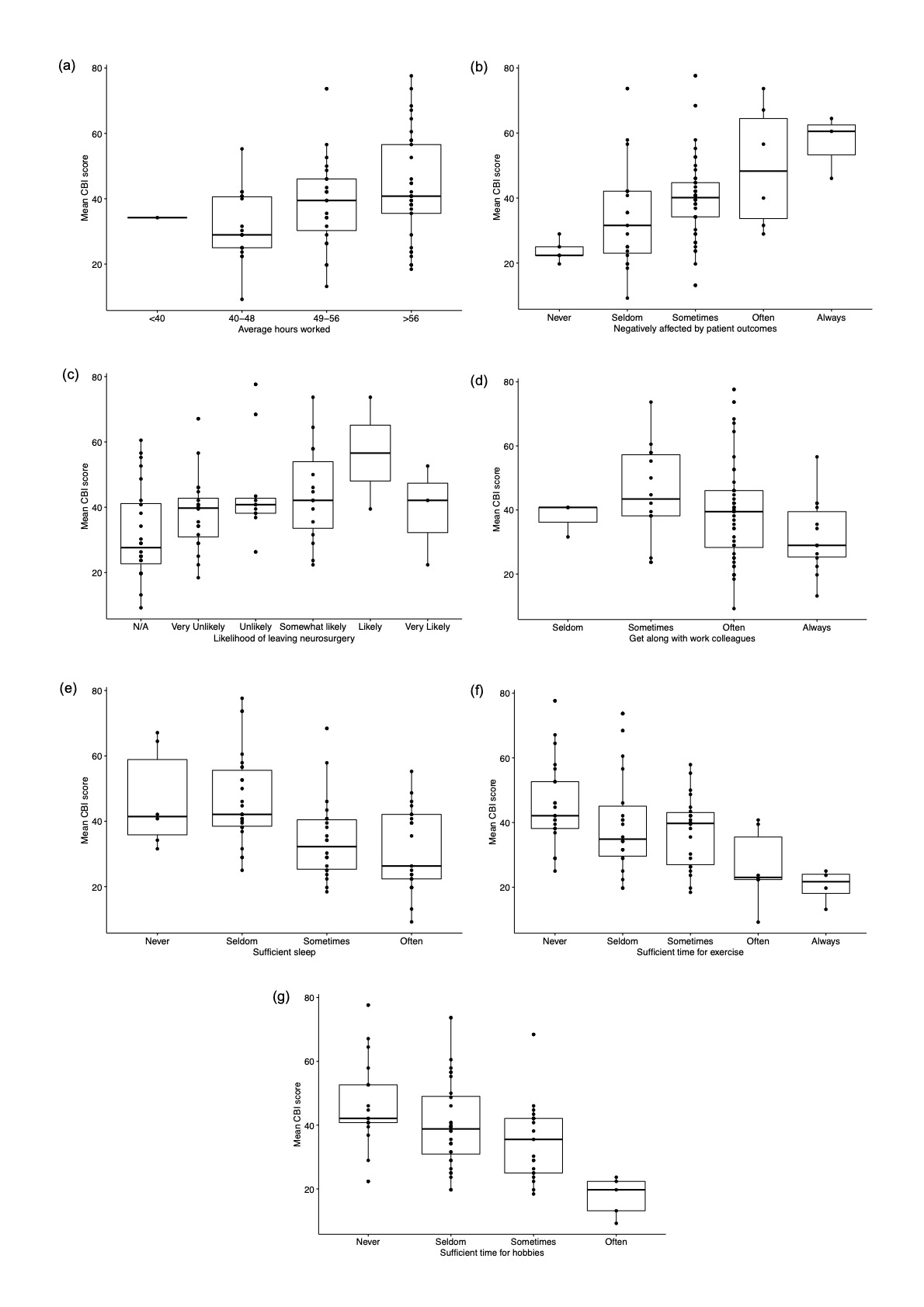

Supplement: Supplementary file 1 — Supplementary file1 (DOCX 214 KB) [file 701_2021_4873_MOESM1_ESM.docx]
